# A mineralogical study in contrasts: highly mineralized whale rostrum and human enamel

**DOI:** 10.1038/srep16511

**Published:** 2015-11-10

**Authors:** Zhen Li, Maisoon AI-Jawad, Samera Siddiqui, Jill D. Pasteris

**Affiliations:** 1 Department of Earth and Planetary Sciences, Washington University in St. Louis, St. Louis, MO, 63130, USA; 2 College of Resources and Environmental Sciences, Nanjing Agricultural University, Nanjing, 210095, China; 3 Institute of Dentistry, Barts and the London School of Medicine and Dentistry, Queen Mary University of London, London, E1 4NS, UK

## Abstract

The outermost enamel of the human tooth and the rostrum of the whale *Mesoplodon densirostris* are two highly mineralized tissues that contain over 95 wt.% mineral, i.e., bioapatite. However, the same mineral type (carbonated hydroxylapatite) does not yield the same material properties, as revealed by Raman spectroscopy, scanning electron microscopy, electron microprobe analysis, and synchrotron X-ray diffraction analysis. Overall, the outermost enamel of a tooth has more homogeneous physical and chemical features than the rostrum. Chemical comparison of rostrum and enamel shows bioapatite in the rostrum to be enriched in Na, Mg, CO_3_, and S, whereas the outermost enamel shows only a slightly enriched Cl concentration. Morphologically, mineral rods (at tens of μm scale), crystallites and prisms (at μm and sub-μm scale), and platelets (at tens of nm scale) all demonstrate less organized texture in the rostrum than in enamel. Such contrasts between two mineralized tissues suggest distinct pathways of biomineralization, e.g., the nature of the equilibrium between mineral and body fluid. This study illustrates the remarkable flexibility of the apatite mineral structure to match its chemical and physical properties to specific biological needs within the same animal or between species.

Bone and tooth are the two major mineralized tissues of vertebrates, both consisting of a nanocomposite of protein and inorganic calcium phosphate^1^,^2^,^3^. Typical bone contains 45 to 65 wt.% mineral, 40 to 20 wt.% organic material, and the reminder water^2^,^4^,^5^. Dentin in vertebrate teeth contains ~70 wt.% mineral, 20 wt.% organic material, and 10 wt.% water^6^. In contrast to the above are what might be considered from a materials science viewpoint to be the highly mineralized analogs of dentin and bone, namely tooth enamel and the hypermineralized rostrum of certain species of whale.

Like many species of whales and dolphins, the whale *Mesoplodon densirostris* has an elongated facial projection called a rostrum. What is unusual in this whale species is that the rostrum of the adult male is composed of highly mineralized bone, containing ~96 wt.% mineral, which is the densest bone so far recorded^4^,^7^,^8^,^9^. Enamel, unlike the highly mineralized rostrum, is universal in all toothed animals and has a mineral content >90 wt.%. The outermost region (several tens of μm thick) of the enamel contains 95–97 wt.% mineral^3^,^6^. The outermost enamel and the hypermineralized rostrum are hence the two most highly mineralized tissues in vertebrate bodies.

The inorganic compounds in the rostrum and enamel are similar to the mineral hydroxylapatite, Ca_10_(PO_4_)_6_(OH)_2_. The biologically precipitated form of apatite, i.e., bioapatite, is actually a type of carbonated hydroxylapatite (CHAP, carbonate mostly substituting for phosphate) with considerable depletion of hydroxyl^10^,^11^,^12^,^13^. In addition to the substitution of CO_3_^2−^ for PO_4_^3−^ in bioapatite, Na, Mg, or other minor/trace elements can substitute for Ca cations. The much more complex bioapatite formula is therefore similar to (Ca,Mg,Na)_10−x_[(PO_4_)_6−x_(CO_3,_ HPO_4_)_x_](OH)_2−x_^10^,^14^.

Organic matrix-mediated biomineralization is the process understood to govern tooth and bone formation. Biologically precipitated crystals grow on a framework of hydrophobic organic matrix, regulated by genetically controlled hydrophilic acidic proteins that promote crystallization. In enamel, the non-collagenous protein amelogenin makes up >90% of the organic component^3^,^6^,^15^. Enamel mineralization starts at the enamel-dentine junction, guided by enamel proteins (predominantly amelogenin) coating the secretory surface of the ameloblast plasma membrane. During the secretory stage, enamel starts growing in elongated ribbons oriented perpendicular to the mineralization front. Recent studies suggest these initial enamel ribbons are amorphous calcium phosphate (ACP) whose size, shape, and spatial organization are defined before they crystallize into carbonated hydroxylapatite^16^,^17^,^18^. Ameloblasts develop Tomes’ processes, which change the topographical contour of the mineralization front and give rise to the final rod and interrod hierarchical organization of enamel. Once the full thickness of the enamel layer is reached the ameloblasts transition in size and architecture into maturation-stage ameloblasts. The final stage of formation is enamel maturation, which involves widening and thickening of the ribbon-like crystallites while enamel proteins removed by the KLK4 enzyme make space for the thickening mineral crystallites^19^. The complex and dynamic processes of amelogenesis are responsible for the final hierarchical, highly mineralized enamel tissue with structural and mechanical properties necessary for its function.

Bone’s mineralization is regulated by collagen, non-collagenous proteins, and bone cells. The mineralization processes can be summarized as follows^3^,^5^,^20^,^21^,^22^,^23^: (1) osteoblasts secret collagenous proteins; (2) collagen molecules form a fibrous framework, which usually grows parallel to the long axis of the bone; (3) bioapatite nanocrystals are initiated in periodic intrafibrillar gaps or in channels between collagen fibrils; (4) initial nanocrystallites are stabilized and then enlarged; (5) the collagen framework remains in bone even after mineral maturation. In further contrast to enamel, bone is a dynamic tissue, which is constantly remodeled (undergoing dissolution and reprecipitation) by osteoclast and osteoblast cells. Remodeling produces secondary osteons of cylindrical shape, which are widely distributed in bone. The highly mineralized whale rostrum is recognized to be densely populated by mineral-rich, collagen-depleted secondary osteons throughout its entire extent^4^,^7^,^8^,^24^,^25^.

The aim of this paper is to step back from the effects of the hierarchical organization and instead to focus in detail on the mineralogical features of these two highly mineralized tissues, rostrum and enamel (primarily the outermost region). They have developed independently and yet have almost exactly the same degree of mineralization. The size, shape, orientation, chemistry, and degree of crystallinity of the two sets of bioapatite crystals are investigated. Understanding their mineralogical contrasts in detail may make possible, through synthetic biomimetic pathways, controlled mineralization for therapeutic uses (e.g., enamel and bone re-growth).

## Results

In order to clarify the similarities and differences in the physical appearance of outermost enamel and rostrum at various spatial scales, the term crystallites and prisms will be applied to enamel and rostrum respectively for features at the sub-micron length scale. The terms rod and interrod will be used to define features at the micrometer length-scale (bundles of crystallites). For ease of comparison, this strictly morphological usage on the finest scale coincides with the dental technical use of these terms.

### Morphology and sizes of bioapatite

**Figure 1:**
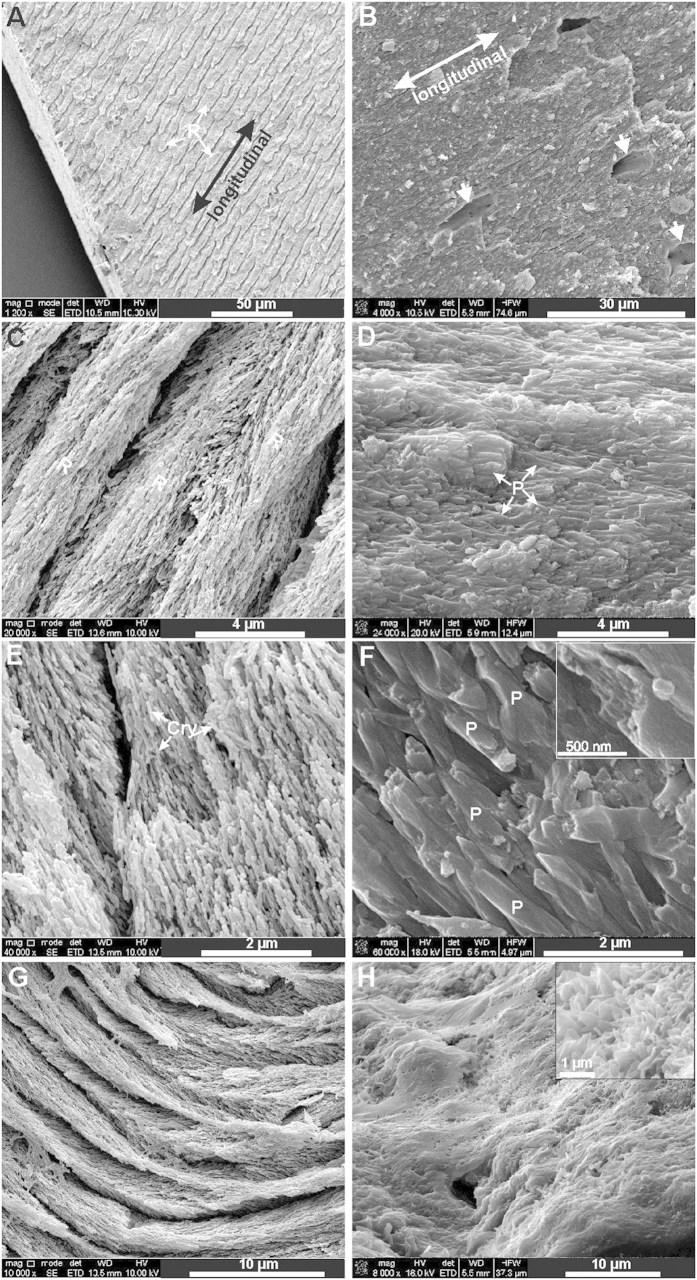
FE-SEM images of human enamel (A,C,E,G) and the rostrum (B,D,F,H). Images (**A**–**F**) are longitudinal views and images (**G**–**H**) are transverse views (R: rods; Cry: crystallites; P: prisms). Arrows in (**B**) indicate lacunae in the rostrum. An enlargement in (**F**) shows how platelets organize to form a prism. An enlargement in (**H**) shows the sharp corners of the blade-like platelets.

At the scale of tens of micrometers in the outermost enamel, are rods with widths of several micrometers and lengths of ~30–40 μm separated by regions of less organized mineral (less than 1 μm in width) (Fig. 1A,C). These rods are clearly bundles of exactly parallel crystallites oriented in the longitudinal direction, as viewed under FE-SEM (field-emission scanning electron microscopy) (Fig. 1A, E). In contrast, the rostrum does not show any distinguishable rod/interrod features nor bundles of crystallites at this scale (Fig. 1B).

At the micrometer scale (Fig. 1C,E vs. Fig. 1D,F), the morphologic features in the rostrum and enamel show even greater difference. The enamel rods appear to consist of thick needle-like crystallites (Fig. 1C,E), whereas at this length-scale the rostrum reveals elongated tablet-like prisms (Fig. 1D,F), which are approximately 1 μm × 0.5 μm in size. Their small angular deviations, however, contrast with the strict parallelism of needle-like crystallites seen in enamel (Fig. 1C,D). Distinctive sizes and shapes of the mineral crystallites and prisms in the two highly mineralized materials are displayed clearly at the micrometer scale (Fig. 1E,F); the rostrum tablet-like prisms are larger than the enamel crystallites in width, but are shorter than the enamel in length^8^. In addition, the enamel crystallites are uniform in size at the micrometer and sub-micrometer scale, compared to the variable sizes of prisms in the rostrum (Fig. 1C vs. D). Figure 1F also illustrates the “irregular” shapes of prisms in the rostrum: long lateral edges of these prisms are not straight like those in enamel crystallites, but usually curved. Only at an even higher resolution (Fig. 1F insert) do the rostrum prisms show themselves to consist of even thinner platelets, which may account for the irregular outline of the prisms (which are organized aggregates of platelets).

In Fig. 1G, individual “layers” can be identified in the transverse section of the enamel. The alignment of such bundles of crystallites in enamel actually forms a well-organized, solid scaffold structure of rod/interrod enamel^26^. The transverse section of the rostrum also shows layers within the stacks of platelets (Fig. 1H). However, its layer structure is not as well-organized as that of enamel. The blade-like edges of mineral platelets in the rostrum are aligned nearly parallel (see the enlargement in Fig. 1H), consistent with the stacks of plates in the longitudinal view (see the enlargement in Fig. 1F).

### Raman spectroscopy

**Figure 2:**
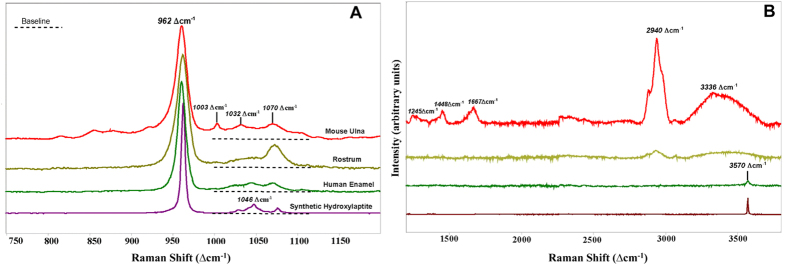
Raman spectra of rat ulna, whale rostrum, human enamel (outermost area), and OHAP. All peak strengths were normalized to the intensity of the 960 Δcm^−1^ peaks. (**A**) 750–1200 Δcm^−1^, (**B**) 1200–3800 Δcm^−1^.

Raman spectra of the rostrum, outermost human enamel, and bone from the rat ulna, show peaks for the mineral and organic phases (where present). In Fig. 2A, the dominant peak for the mineral phase in all samples is a P-O symmetric stretch at ~960 Δcm^−1^, which together with its other peaks identifies the phosphate phase in all bone and enamel materials as apatite^27^,^28^,^29^,^30^. Compared to that of the rostrum and human enamel, the P-O stretch of the rat bone is slightly downshifted and that of the synthetic OHAP (hydroxylapatite) slightly upshifted. These (small) differences in P-O position reflect differences in the chemical environment of the phosphate in these apatite phases, i.e., small compositional differences among the types of apatite.

The widths of the 960 Δcm^−1^ peaks indicate the degree of atomic order within the structure of the crystallites: the wider the peak, the more atomically disordered the material is^8^,^27^. Such disorder is one component of the property often referred to as crystallinity. The maximum value of FWHM (full width at half maximum) of the ~960 Δcm^−1^ band is considerably larger in the normal bone, rostrum, and enamel than in synthetic OHAP (FWHM = 5.4 cm^−1^). Thus, all three bioapatites are atomically more disordered than synthetic hydroxylapatite. Enamel (FWHM = 13 cm^−1^) contains the most atomically ordered bioapatite, compared to the rostrum (FWHM = 15 cm^−1^) and normal bone (FWHM = 16 cm^−1^).

The 1070 Δcm^−1^ peak represents a combination of the ν_3_ vibrational mode of PO_4_ and the ν_1_ C–O stretching vibration of CO_3_^29^. This peak indicates carbonate substitution for phosphate in bioapatite. The intensity of the rostrum’s 1070 Δcm^−1^ peak is much stronger than that of the human enamel: with a calculated carbonate concentration of 8 wt.% in the rostrum vs. 3 wt.% in enamel apatite based on a calibration in our laboratory^31^. The rat bone has a carbonate content of ~6 wt.%, between that of the rostrum and human enamel. In addition, rat bone has the widest FWHM for the 1070 Δcm^−1^ peak (Fig. 2A), consistent with its widest FWHM for the 960 Δcm^−1^ band. Therefore, the bioapatite in both highly mineralized tissues is atomically more ordered than that in normal bone.

**Figure 3:**
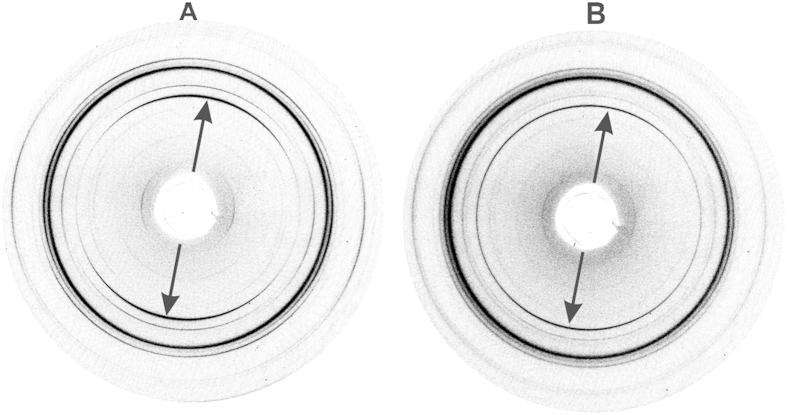
Debye rings of two spot analyses (1 mm apart) on the longitudinal section of the rostrum. The stronger (**A**) and weaker (**B**) intensity of the 002 reflection are indicated by arrows. The variation in the intensity around the Debye ring of the 002 reflection in the left image indicates preferred orientation of the crystallites.

Most of the organic matrix in bone is collagen. The ν(C-C) aromatic ring stretch at 1003 Δcm^−1^ for the phenylalanine component of collagen was detected only in the rat bone (Fig. 3A). This peak occurs in all normal bones^28^,^30^. In addition, amide peaks at 1245 Δcm^−1^ and 1667 Δcm^−1^ 29 and a peak assigned to C-H bending at 1448 Δcm^−1^ 28,29 are present in rat bone (Fig. 2B) and confirm its organic matrix.

There is an envelope of peaks centered at about 2940 Δcm^−1^ that indicate less specific C-H stretching vibrations of organic matter^28^,^32^. The dominant peak at ~2940 Δcm^−1^ is very strong in normal bone matrix, but extremely weak to undetected in the rostrum and enamel. Based on the strength of the 2940 Δcm^−1^ envelope of peaks (Fig. 2B) and a previous calibration^8^, the rostrum’s organic content (excluding vascular areas) is about 4 wt.% and definitely higher than that of the enamel.

The spectrum of OHAP shows a strong peak at 3570 Δcm^−1^, which is the symmetric O-H stretch of hydroxyl in apatite^29^. This peak is weak in the enamel but not detected in either rat bone or the rostrum (Fig. 2B), which is typical in Raman and IR analyses of bone^13^.

### Mineral orientation and sample texture determined by synchrotron X-ray diffraction

Synchrotron X-ray diffraction analyses elucidate preferred orientation of mineral crystallites in the enamel and rostrum samples. The variation in intensity around the Debye ring of the 002 Bragg reflection was used to evaluate the extent of alignment, i.e., preferred orientation of crystallites. Figure 3 shows two typical 2D diffraction patterns from the whale rostrum. They illustrate the range of variation in alignment, i.e., “sample texture,” with respect to the (002) plane at two positions showing maximum crystallite alignment (Fig. 3A, see arrow) and minimum crystallite alignment (Fig. 3B), which are spaced 1 mm apart within the transverse section of the rostrum.

**Figure 4:**
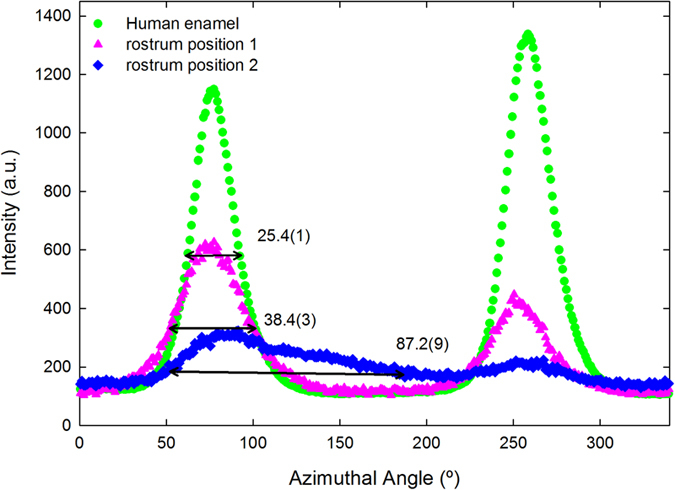
The intensity of diffraction versus azimuthal angle for the 002 reflection of apatite crystallites. The analyses were performed in a longitudinal section of human enamel (outermost region) and for two points in the longitudinal section of the rostrum.

Figure 4 shows the result of integrating the intensity of the 002 reflections over 360^o^ in a narrow band containing just the 002 reflection (e.g., an annular ring in Fig. 3A,B); these values were plotted versus the azimuthal angle for three individual spots. Figure 4 shows for each of the three traces two pronounced alignment maxima separated by approximately 180^o^, for one position in the outermost enamel and the two positions in the rostrum that are shown in Fig. 3B. The wider the FWHM of the azimuthal traces, the less is the preferred orientation (often referred to as “texture”) of crystallites in the sample. The whale rostrum is not as textured as outer enamel based on FWHM values: 38.4(3)^o^ (rostrum, pink) vs. 25.4(1)^o^ (enamel, green). Therefore, the outermost enamel shows greater preferred orientation of its bioapatite crystallites. Figure 4 also shows strong variation in the degree of crystal orientation within the rostrum, as illustrated by the much greater FWHM of 87.2(9)^o^ of the second position (blue).

**Figure 5:**
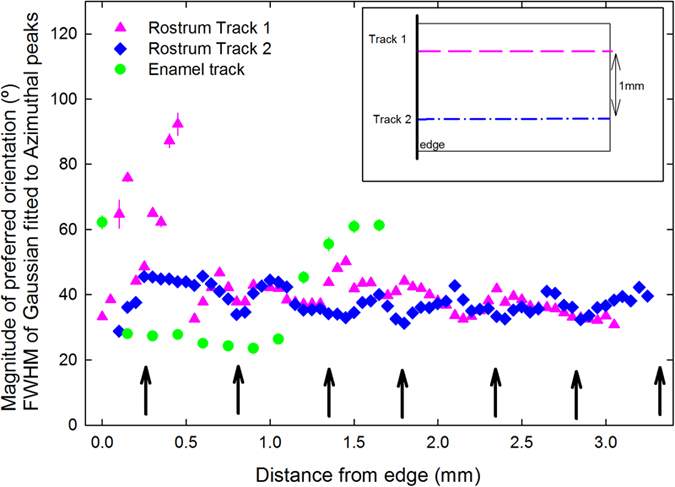
FWHM of the 002 azimuthal peaks. The FWHM values are shown as a function of position (distance from edge) for two tracks within the longitudinal section of the whale rostrum, and for one track through healthy human enamel (from surface to EDJ). Repeated cycles of increase and decrease in magnitude of preferred orientation are shown by arrows for Track 2. Track 1 and Track 2 are 1 mm apart (as shown in the diagram inset).

The FWHM was determined as a function of position for one track in the enamel, and for two tracks in the rostrum sample; both samples are longitudinal sections. For the rostrum, the distance was measured from the edge of the specimen piece, and for the enamel it was from the outermost surface (in the direction toward EDJ). The FWHM versus distance is presented in Fig. 5, where the magnitude of preferred orientation at each point is an average over a volume of 50 × 50 × 200 μm. Figure 5 shows a periodic increase and decrease in magnitude of preferred orientation with a repetition every 0.3–0.5 mm across the longitudinal section of the rostrum. These length-scales suggest that each repetition cycle covers one or two osteons, whose diameters are 200–300 μm. The relatively small values of FWHM indicate that the crystallites are relatively well organized/aligned inside of osteons. The relatively small variation in FWHM values across the full length of track 2 and most of track 1 indicates the repetition of the pattern of the osteons. The sharp increase in values on the left end of track 1 in Fig. 5 demonstrates weaker preferred orientation than in most areas. Such lack of order may be due to the occurrence of primary osteons (with less well aligned collagen and crystallites) at this end of the track. The major part (~1 mm thick) of the enamel shows low and relatively constant FWHM values, but the values increase sharply near the EDJ. The outer 50–100 μm layer of enamel has the highest FWHM. This is a region that is reported to be “prismless” in the technical dental sense, and therefore appears to have less crystallite organization^33^. It is also instructive to note that data for normal bone cannot even be plotted on Fig. 5. The bone crystallites show so little alignment that the 002 Bragg reflection does not have regular variations in its azimuthal intensity.

### Electron microprobe (EMP) analyses

The EMP technique gives precise *in-situ* chemical analyses of the rostrum and outermost region of the enamel, as the analyst can avoid vasculature in the rostrum and measure only areas above the enamel-dentin junction in enamel. Human enamel shows an average Ca/P ratio of 1.63, which is significantly less than both the 1.71 of the rostrum and 1.67 of OHAP (Table 1).

Compared to the rostrum, the outermost enamel shows lower Na, Mg, S, and F concentrations. The average composition of the rostrum has 1.5 wt.% Na_2_O and 0.8 wt.% MgO (Table 1), which are more than double the values of 0.7 wt.% Na_2_O and 0.3 wt.% MgO in the enamel. In addition, F and S are almost absent in the enamel, whereas the rostrum has 0.5 wt.% SO_3_ and 0.4 wt.% F. Chlorine is the only minor element that is more abundant in the enamel than in the rostrum. Potassium is extremely low in both samples. The weight percent sum of the analyzed inorganic components of the enamel is significantly higher than that of the rostrum, which in large part is due to different carbonate contents in the two materials: ~3 wt.% (enamel) vs. ~8 wt.% (rostrum)^34^,^35^. Because carbon was not analyzed directly in these samples, the carbonate-rich rostrum mineral will show an enhanced mass deficit. The outermost enamel and the rostrum therefore both have mineral contents of 96–97 wt.%.

## Discussion

Their similar mineral contents (96–97 wt.%) and mineral species (CHAP) make the rostrum and outermost enamel two comparable highly mineralized materials. Even though rare specimens of the whale rostrum and typical specimens of tooth enamel share approximately the same extremely high degree of mineralization, the bioapatite mineral component differs between them in composition, size, shape, and preferred orientation.

Compared to the rostrum, the chemistry of enamel is closer to that of standard OHAP in its minor/trace elements and carbonate. The rostrum and outermost enamel show much more crystallite alignment than in typical bone. Synchrotron X-ray micro-diffraction shows a gradual increase in FWHM of 002 azimuthal peaks of crystallites from the outermost region (the subject of the current study) downward to the EDJ^36^. Therefore, only the outermost region of the enamel has a degree of preferred orientation of crystallites (that form mineral rods at micrometer scale) that equals or exceeds that of the rostrum. The outermost enamel also has a higher degree of atomic order within its mineral (as assessed by FWHM of Raman peaks) and larger size of crystallites (assessed by XRD in Rogers and Zioupos, 1999).

Mineralogical properties are interconnected to each other in the rostrum and enamel: (1) greater crystal perfection (which also typically accompanies lower carbonate concentration) gives greater reproducibility in characteristics such as size and shape. The crystallites in the outermost enamel are larger than those in the rostrum, but smaller than those in many types (depending on formation conditions) of synthetic OHAP^4^. The enhancement in crystallite perfection and size may account for the greater preferred orientation of crystallites in the outermost enamel with respect to the rostrum, as confirmed by the FWHM values in Fig. 5; (2) variations in the FWHM values for (002) azimuthal orientation in enamel correlate with variations in the chemistry of enamel^37^,^38^. Furthermore, the larger deviation of rostrum’s chemistry (see Table 1) could also be related to its larger FWHM values; (3) both the rostrum’s greater incorporation of trace/minor elements and its higher carbonate content could contribute to the lower degree of atomic order of the rostrum’s bioapatite^34^,^39^; (4) These mineralogical properties probably determine their mechanical properties, for example, the Vicker’s hardness is 220 for hypermineralized rostrum^4^ vs. 350–420 for enamel^40^.

A mineralogical view could shed light on, at least part of, two distinct pathways of rostrum’s and enamel’s formation. The apatite structure is capable of accepting a wide range of chemical substitutions. Such chemical substitutions change the properties of the mineral, such as its size, shape, and solubility^2^,^10^. Mineralogical control is imposed on the nature and extent of chemical substitutions in the bioapatite by the chemistry (composition, pH) of the solution from which the apatite precipitates. In addition, the cells and biochemical compounds in the tissues that give rise to the rostrum and enamel mineral can control the composition of the biomineral, but only to the extent that they control the chemistry of the apatite-precipitating fluid. The consequence of the tight linkage between the composition of a mineral (apatite) and its physical-chemical properties is that a desired change in shape, size, solubility, etc. is accomplished most straightforwardly by changing the precipitating fluid’s composition. However, attainment of one set of desired characteristics brings with it all the other consequent features. For instance, platelet-shaped apatite is achieved by elevating the carbonate concentration in the apatite-precipitating fluid (even in the absence of any organic component), whereas apatite prisms elongated parallel to their crystallographic c-axes form only in solutions with much lower carbonate concentration^41^.

This issue of probable difference in the composition of the fluid from which rostrum apatite and enamel apatite grew may reflect the difference in matrix-mineral relations between rostrum and enamel. As described in the introduction, enamel mineralization does not occur within an existing, assembled organic matrix, as occurs in bone formation. Rather, enamel’s mineralization occurs simultaneously with the self-assembly of the protein (amelogenin). As further noted by Margolis *et al.* (2014), the timing of the activity of mineral regulators (such as crystallization inhibitors) during biomineralization is critical to the nature of the mineral^42^. The timings of the beginning and the duration of matrix-mineral interactions clearly are very different for enamel and hypermineralized bone, even though both materials ultimately attain about the same degree of mineralization.

The biochemistry and cellular activity within the rostral and dental tissues cannot separate the physical functionality of the bioapatite from its inherent chemistry. If platelet-shaped apatite is needed to create the nanocomposite bone (as in the rostrum), then that apatite will contain about 5–8 wt.% carbonate^29^,^43^. If strong, hard apatite of relatively low solubility is required (as in enamel), then it will contain about 2.5–4 wt.% carbonate^34^. It has been reported that the incorporation of carbonate into the lattice of apatite leads to a smaller crystallite size^2^,^10^,^39^. Therefore, the lower-carbonate (compared to the rostrum) concentration of bioapatite in the outermost enamel probably permits its larger crystal sizes.

## Conclusions

The rostrum and outermost enamel therefore share several important mineralogical features in mineral type, mineral content, and organized longitudinally oriented prisms/rods. However, the physical and chemical properties of the two highly mineralized tissues also show significant differences in degree of crystallinity, specific mineral composition, and degree of preferred orientation of prisms. Compared to the rostrum, the outer tooth enamel is a more homogeneous tissue with greater mechanical strength, lower incorporation of non-apatitic ions, and higher degree of preferred orientation of crystallites. The difference in composition of the two highly mineralized tissues could reflect differences in equilibrium between mineral and appropriate body fluid composition, blood plasma vs. saliva; therefore understanding the mineralogical differences between the rostrum and enamel can help guide future studies about the biological mechanisms of mineralization in bone and tooth.

## Materials and Methods

### Preparation of the whale rostrum, human enamel, and rat ulna bone

Rostrum material from an adult male whale (#1922–143) of species *Mesoplodon densirostris* was obtained from the Muséum National d’Histoire Naturelle in Paris, France. Portions of this same sample have undergone previous histological, chemical, and mechanical investigation^8^,^35^. For the present study, one 4 × 2 × 1 cm block was sawn from the original sample. A portion of this block was then embedded in epoxy, ground and polished for Raman spectroscopy and electron microprobe analysis. A 4 × 2 × 0.2 mm longitudinal section was cut from the block and polished for synchrotron X-ray diffraction analysis. Small particles (non-epoxied) released during sample preparation were collected for analysis by scanning electron microscopy.

A healthy human upper 1^st^ premolar was taken with ethical consent from the tissue bank at the Institute of Dentistry, Queen Mary University of London, UK (QMREC2008/57). The informed consent was obtained from all subjects of the premolar. For the synchrotron X-ray diffraction measurements, the tooth was sawn into a 0.2 mm thick section parallel to the bucco-lingual plane using an Accutom-5 saw (Struers Ltd, Ballerup, Denmark) with a diamond-edged blade. The section was polished using silicon carbide paper. For scanning electron microscopy this specimen was then etched for 15 seconds in 35% orthophosphoric acid to remove the smear layer (most in dentin area), then washed with distilled water and left to dry under vacuum overnight.

Ulna bone from a post-pubescent rat and synthetic hydroxylapatite (OHAP) were analyzed by Raman spectroscopy for comparison with the rostrum and enamel. As the example of normal bone in this study, the ulna was fixed and dehydrated in graded alcohols and stored in 100% ethanol, and was sectioned using a diamond-edged blade to produce parallel sections of 50 μm thickness. OHAP powder used in the study is 99.999% pure on a metal basis (Sigma-Aldrich®, St. Louis, MO).

In summary, the rostrum and human enamel were measured using all listed techniques. In distinction to the two primary specimens (rostrum and human enamel), rat ulna and OHAP were measured only by Raman spectroscopy. All samples were obtained and experiments were performed in accordance with relevant guidelines and regulations.

### Instrumentation

#### Electron microprobe analysis

EMP analysis was performed with a JEOL JXA 8200 Superprobe. Carbon-coated polished sections of rostrum and human tooth enamel (outermost region) were studied with an accelerating voltage of 15 kV. Quantitative point analysis was accomplished with a beam current of 25 nA and a beam diameter of 20 μm. The elements F, Na, Mg, P, S, Cl, K, and Ca were selected for quantitative analysis by wavelength-dispersive X-ray spectroscopy (WDS). The calibration standards for the EMP analyses included natural geological minerals: Durango apatite (Ca_10_(PO_4_)_6_F_2_) for Ca, P, and F, albite (NaAlSi_3_O_8_) for Na, synthetic forsterite (Mg_2_SiO_4_) for Mg, anhydrite (CaSO_4_) for S, microcline (KAlSi_3_O_8_) for K, and tugtupite (Na_4_AlBeSi_4_O_12_Cl) for Cl. The spot analyses were randomly distributed throughout the transverse sections of enamel and rostrum.

#### Field-emission scanning electron microscopy

**FE-SEM on the rostrum was applied using an FEI NOVA 2300 system. The tooth enamel sample was observed using an FEI Inspect F50 (Oxford Instruments, UK). Both samples were sputter-coated with gold and were analyzed at an accelerating voltage of 5–20 kV.

#### Raman microprobe spectroscopy

Raman spectra were acquired to observe vibrational modes that characterize the rostrum, outermost enamel, normal bone, and OHAP. Analyses were performed with a fiber-optically coupled Raman microprobe (HoloLab Series 5000 Raman Microprobe, Kaiser Optical System, Inc.). The spectral region of 100–4000 Δcm^−1^ was recorded using 532 nm excitation at 10 milliwatts laser power on the sample surface. The diameter of the focused laser spot was ~1 μm for all Raman spectra. Ten point analyses were made randomly on each sample (mineral-rich area) to avoid singularity, and each sample showed homogeneity of mineral peaks.

#### 2D synchrotron X-ray micro-diffraction

The samples were analyzed at the European Synchrotron Radiation Facility (ESRF) on the XMaS (BM28) beamline. An X-ray wavelength of 0.82 Å and a beamspot size of 50 × 50 μm were used. The rostrum and human enamel were mounted in transmission geometry onto a sample holder able to move perpendicular to the X-ray beam in X and Y directions. The region of interest on each specimen was identified using a telescope focused on the center of rotation of the mounted sample, and co-ordinates were identified for tracking analyses. A 2048 × 2048 pixel CCD detector was positioned approximately 200 mm behind the specimen in order to collect 2D diffraction images every 30 seconds^36^. Using the ESRF software package Fit2D, the variation in intensity of the 002 Bragg reflection was plotted as a function of azimuthal angle around the Debye ring. The peaks were fitted with a Gaussian peak-shape, and the FWHM indicates the degree of alignment along the crystallographic c-axis direction.

## Additional Information

**How to cite this article**: Li, Z. *et al.* A mineralogical study in contrasts: highly mineralized whale rostrum and human enamel. *Sci. Rep.* **5**, 16511; doi: 10.1038/srep16511 (2015).

## Figures and Tables

**Table 1:** EMP quantitative analyses on the rostrum (R) and outermost human enamel (E) (N = 5).

#	CaO	P_2_O_5_	Na_2_O	MgO	SO_3_	K_2_O	Cl	F	Ca/P	Sum
E1	51.99	40.56	0.65	0.22	0.01	0.03	0.55	0.03	1.62	93.89
	(0.2)	(0.4)	(3.1)	(5.6)	(124.9)	(15.5)	(1.2)	(43.8)		
E2	51.88	40.36	0.75	0.30	0.02	0.01	0.47	0.00	1.63	93.69
	(0.2)	(0.4)	(2.8)	(4.3)	(32.9)	(39.2)	(1.4)	(1038)		
E3	51.75	40.05	0.81	0.35	0.01	0.01	0.42	0.01	1.63	93.31
	(0.2)	(0.4)	(2.7)	(3.8)	(91.4)	(31.7)	(1.5)	(204.4)		
E4	51.94	40.17	0.76	0.35	0.02	0.01	0.45	0.04	1.64	93.64
	(0.2)	(0.4)	(2.8)	(3.7)	(38.8)	(36.2)	(1.4)	(37.2)		
E5	52.19	40.46	0.69	0.25	0.01	0.01	0.51	0.00	1.63	94.01
	(0.2)	(0.4)	(3.0)	(5.0)	(105)	(30.1)	(1.3)	(−149)		
R1	48.60	35.58	1.40	0.87	0.49	0.03	0.04	0.28	1.73	87.29
	(0.6)	(0.5)	(2)	(2)	(2)	(12)	(7)	(6)		
R2	47.65	35.30	1.44	0.81	0.48	0.02	0.04	0.31	1.71	86.08
	(0.6)	(0.5)	(1)	(2)	(2)	(17)	(8)	(5)		
R3	48.17	35.82	1.50	0.80	0.54	0.02	0.04	0.35	1.70	87.27
	(0.6)	(0.5)	(1)	(2)	(2)	(14)	(7)	(5)		
R4	48.65	35.66	1.66	0.82	0.42	0.04	0.04	0.20	1.73	87.55
	(0.6)	(0.5)	(1)	(2)	(3)	(10)	(8)	(8)		
R5	48.57	36.16	1.41	0.88	0.74	0.03	0.05	0.61	1.70	88.48
	(0.6)	(0.5)	(1)	(2)	(2)	(11)	(6)	(3)		
E	51.95	40.32	0.73	0.29	0.01	0.02	0.48	0.02	1.63	93.71
	±0.16	±0.21	±0.06	±0.06	±0.01	±0.01	±0.05	±0.02	±0.01	±0.27
R	48.33	35.70	1.48	0.84	0.53	0.03	0.04	0.35	1.71	87.33
	±0.42	±0.32	±0.11	±0.04	±0.12	±0.01	±0.00	±0.16	±0.02	±0.86

Average values are indicated in the two bottom rows in the table. The sum of the analyzed weight percents is shown in the last column. All values shown are in wt% except for the Ca/P atomic ratios. Data on the rostrum (average of eight spots on typical areas) is from Li and Pasteris, 2014. Numbers in parentheses show percent relative error.
