# Erratum: A mineralogical study in contrasts: highly mineralized whale rostrum and human enamel

**DOI:** 10.1038/srep46933

**Published:** 2018-01-08

**Authors:** Zhen Li, Maisoon Al-Jawad, Samera Siddiqui, Jill D. Pasteris

Scientific Reports 5: Article number: 1651110.1038/srep16511; published online: 11 10 2015; updated: 01 08 2018

The original version of this Article contained a typographical error in the spelling of the author Maisoon Al-Jawad, which was incorrectly given as Maisoon AI-Jawad. This has now been corrected in the PDF and HTML versions of the Article.

